# Pedicle screw and rod fixation in three cats with caudal lumbar vertebral fracture and luxation: a case report

**DOI:** 10.3389/fvets.2026.1778936

**Published:** 2026-10-26

**Authors:** S. A. M. Aartsen, L. L. van Stee, B. P. Meij

**Affiliations:** Department of Clinical Sciences, Faculty of Veterinary Medicine, Utrecht University, Utrecht, Netherlands

**Keywords:** fracture repair, lumbar fracture cat, lumbar luxation cat, neurologic damage, pedicle screw and rod fixation (PSRF)

## Abstract

**Background:**

Pedicle Screw and Rod Fixation (PSRF) is used for spinal stabilization of human and, more recently, in dogs. The use of bilateral PSRF in cats at the caudal lumbar and lumbosacral regions has not been reported.

**Objectives:**

To describe the outcomes of cats with caudal lumbar vertebral fractures and luxation treated with bilateral monoaxial PSRF surgery.

**Animals:**

Three cats with traumatic caudal lumbar vertebral fractures and luxation treated with bilateral PSRF (2016-2018).

**Methods:**

Short case series. A retrospective analysis of medical records of cats treated with PSRF was performed. Data collected included clinical signs, neurological examination, pre-operative radiographs, and pre-operative CT scan. Post-operative neurological examination and radiographs 6-7 weeks post-operative were conducted. All radiographs and CT scans were reviewed by a board-certified veterinary radiologist. Inclusion criteria were cats presented to the clinic with thoracolumbar fractures and luxations without comorbidities. All cats were examined by a veterinary surgeon or surgery resident before surgery, immediately post-operative, 2 out of 3 cats presented 2-3 weeks post operative and all cats presented 6-7 weeks post-operative. Outcome assessment included comparing the neurological grade of the cats before and after surgery and the feasibility of placing the PSRF system in the vertebra of the cats.

**Results:**

All cats underwent bilateral PSRF for stabilization of the caudal lumbar vertebrae to decompress and stabilize the spinal cord and cauda equina. The first cat presented with a comminuted fracture and luxation of L6 neurological grade III according to the modified Griffith Sharp-Wheeler scale and improved to grade 0 neurological status after surgery. The second cat had a caudal comminuted vertebral body fracture of L7 with minor dorsal dislocation and improved after surgery from neurological grade IV to grade III post-operatively. The third cat presented with a complete L5-L6 luxation with multiple fractures and displaced fracture fragments and a neurological grade V; no improvement was observed following surgery.

**Conclusions and clinical importance:**

PSRF is a technically feasible surgical option for the treatment of vertebral fractures and luxations in cats; emphasizing the potential of the PSRF system in cats warrants further exploration.

## 1 Introduction

Vertebral fractures and luxations are devastating conditions (1) that can result in a variety of severe clinical signs, including ataxia, paraparesis and paralysis, with or without deep nociception (2, 3). Due to fractures and luxations of the vertebra, the spinal cord can be traumatized and compressed by misalignment of vertebrae causing severe progressive neurological signs and deficits. Treatment includes conservative medical treatment with adequate analgesia and cage rest or surgical strategies. Surgical treatment consists of realignment and stabilization of the spinal column and decompression of the spinal cord. A variety of surgical techniques can be used to stabilize vertebral segments, including the use of tensions bands or screws and polymethylmethacrylate (PMMA) fixation (4, 5), spinous process plating, ventral body plating, modified segmental spinal instrumentation, and external skeletal fixation (6–10, 34). Pins and screws with PMMA are the most frequently used because of their effectiveness for spinal stabilization, although iatrogenic injuries have been reported (4, 5). The current standard of care for vertebral fractures in cats is mostly defined by availability of implants and surgical preference. Most commonly the surgical technique involves segmental fixation using pins or screws and PMMA, although no clear consensus exists regarding treatment of caudal vertebral fractures and luxations in cats. Pedicle Screw and Rod Fixation (PSRF) has been described for treatment of various vertebral diseases in humans (11) and, more recently, in dogs to stabilize the lumbosacral segment and decompress the cauda equina (12–15).

The goal of PSRF is to stabilize the affected spinal unit(s) to facilitate fracture healing or spinal fusion across one or more spinal segments, stabilizing the decompressed segment and mitigating the development of future degenerative changes. The use of PSRF in cats has recently been reported for the thoracolumbar region (16, 17), but not for caudal lumbar and lumbosacral regions. Only very recently a cadaveric study was reported describing a lumbosacral junction probing technique for implant corridors in cats (18). Clinical experience with a polyaxial screw rod system in the thoracolumbar region in cats has only recently been reported (16); in that study most cats (66%) were successfully treated with unilateral polyaxial screws. Eleven of the 16 cases showed improvement of neurological grading, while no improvement was observed in five cases. The clinical outcome was excellent in four cases, functional in six cases and poor in five cases. Surgical decompression combined with PSRF has been widely used in humans to successfully treat degenerative disc disease, spinal stenosis and spinal instability (11, 19, 20). In dogs, PSRF in combination with discectomy has been successfully used as a method to treat degenerative lumbosacral stenosis (12, 14, 15, 21–23). In veterinary literature, the use of PSRF has been biomechanically and clinically evaluated in dogs (13, 14).

The aim of the present study is to describe the surgical technique and outcome in three cats with traumatic caudal lumbar vertebral fractures and luxations (Table 1) treated with a monoaxial PSRF system to stabilize the vertebral column.

**Table 1:** Overview of the patients, their diagnosis and the implants used and pre-operative CT measurements with width (mm) of pedicles on transverse multiplanar reconstruction (MPR) images of vertebra.

Case no	Gender	Age	Body weight (kg)	Breed	Imaging	Diagnosis	Fixation PSRF	PSRF screws	Rods	Point of measurement	L4 left (mm)	L4 right (mm)	L5 left (mm)	L5 right (mm)	L6 left (mm)	L6 right (mm)	L7 left (mm)	L7 right (mm)	S1 left (mm)	S1 right (mm)	Mean (mm)	Mean widths (mm)
																						
1	FC	1yr 3mo	3.5	Eur SH	RX, CT	Fx L6	L6-L7	4 × 3.5: 2× L6 (16 mm), 2× L7 (14 mm)	2x 4.0 (30 mm)	entry width					3.65	3.96	4.11	3.65			3.8425	
										middle width					2.28	2.28	3.04	2.74			2.585	3.36
										end witdth					3.21	3.19	3.95	4.26			3.6525	
										length					11.4	11.4	11.4	11.3			11.375	
2	F	8 mo	3.4	Dom SH	RX, CT	Fx L7	L7-S1	4 × 3.5: 2× L7 (16 mm), 2× S1 (16 mm)	2x 4.0 (40 mm)	entry width							3.36	3.7	4.36	4.69	4.0275	4.42
										middle width							2.44	2.44	5.33	5.5	3.9275	
										end witdth							3.95	3.61	6.71	6.95	5.305	
										length							12.2	11.9	9.54	8.89	11.21333333	
3	MC	6yr 7 mo	4.3	Eur SH	RX, CT	Fx/lux L5-L6	L4-L6	4 × 3.5: 2× L6 (12 mm), 2× L4 (12 mm)	2x 4.0 (50 mm)	entry widthy	3.39	3.15			3.53	3.65					3.43	
										middle width	2.02	1.89			2.64	2.4					2.2375	2.98
										end witdth	3.29	2.92			3.52	3.4					3.2825	
										length	11.3	11.2			10.8	10.8					11.025	

Pre-operative CT Measurements were done on multiplanar reconstruction (MRP) in Radiant of the lengthe and of the width of (1) entry (top, dorsal, transition to facet), (2) middle, and (3) end (ventral, link to vertebral body) part of the left and right pedicle to be captured with the planned pedicle screw.

FC: female castrated, F: female, MC: male castrated, Eur SH: European Shorthair, Dom SH: Domestic Shorthair, Rx: radiographs, CT: computed tomography, Fx: fracture, lux: luxation.

## 2 Case descriptions

Measurements for surgical planning were performed in silico on the pre-operative CT scans using RadiAnt DICOM Viewer (Medixant). Multiplanar reconstructions (MPRs) were produced and the crosshair of the MPR was centered on the planned pedicle in all three cross sections (transverse, sagittal, axial). The width of the pedicle was measured at 3 levels: (1) dorsal (entry point), (2) halfway at midlevel of spinal canal, (3) ventral at level transition to vertebral body (Figures 1A, B). In addition, the dorsoventral length of the screw corridor was measured including the pedicle from dorsal entry point to ventral exit point on the vertebral body (Figures 1A,B). The mean (range) width of the pedicles was 3.4 mm (2.6–3.8 mm) for case 1, 4.4 mm (3.9–5.3 mm) for case 2, and 3.0 mm (2.2–3.4 mm) for case 3. The mean length of the screw trajectory was 11.4 mm for case 1, 11.2 mm for case 2, and 11.0 mm for case 3. The details of the pre-operative CT measurements of the pedicles on MPR images are presented in Figures 1A,B; Table 1. The monoaxial pedicle screw (Vertebral-Fix, Orthopedaesian), illustrated in Figure 2D, has a core diameter of 2.0 mm and a thread diameter of 2.5 mm.

**Figure 1:**
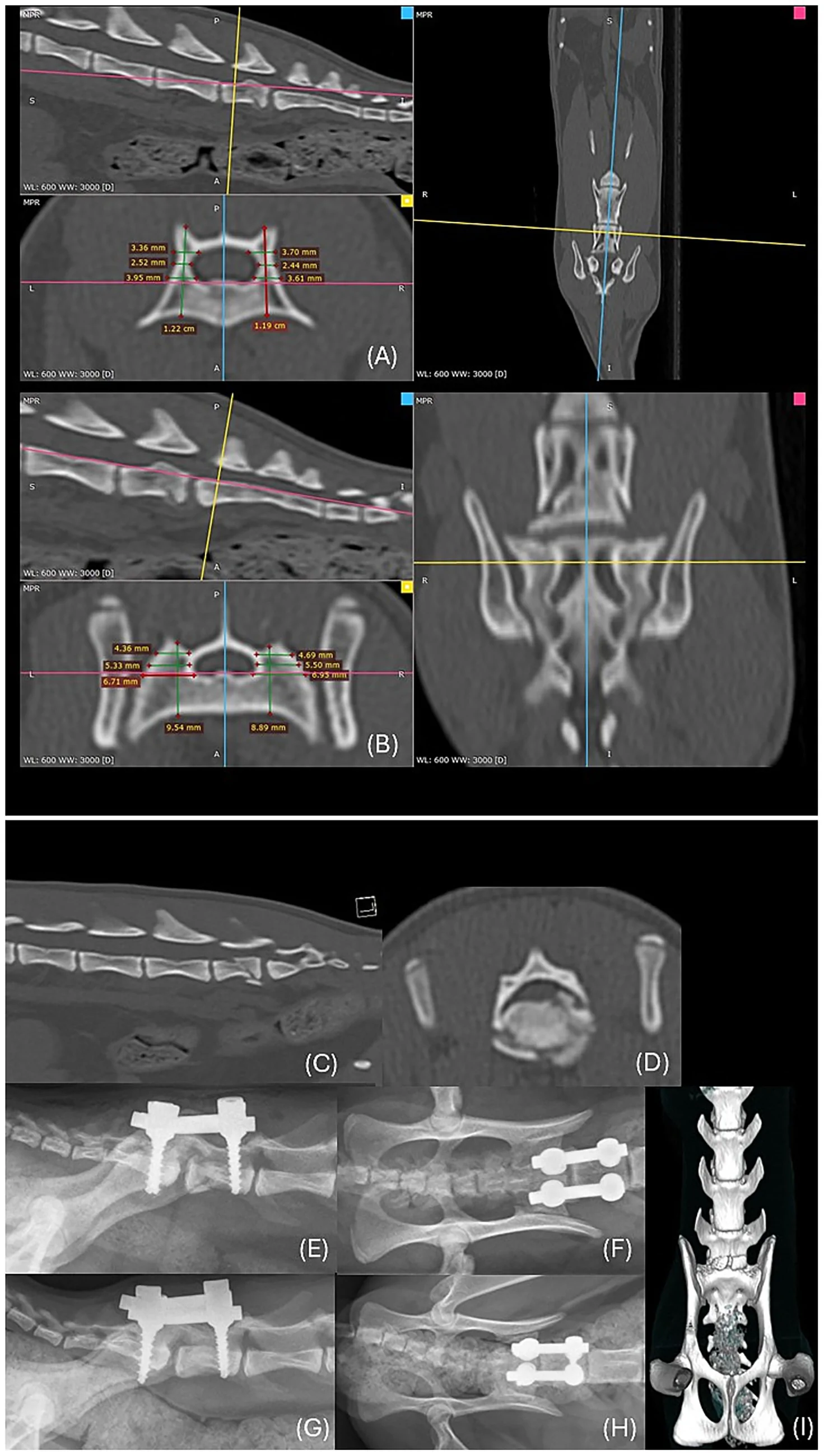
Case 2: radiographs and CT. Multiplanar reconstruction (MPR) in Radiant, CT measurements were done of the width of entry [top, dorsal, transition to facet, middle, end (ventral, link to vertebral body)] part of the left and right pedicle to be captured with the planned pedicle screw. **(A)** Measurements of L7 of case 2. **(B)** Measurement of S1 of case 2. **(C)** Pre-operative longitudinal CT scan case 2. **(D)** Pre-operative saggital CT scan case 2. **(E)** Post-operative right lateral radiograph case 2. **(F)** Post-operative ventrodorsal radiograph case 2. **(G)** 6 weeks post-operative right lateral radiograph case 2. **(H)** 6 weeks post-operative ventrodorsal radiograph case 2. **(I)** 3D reconstruction of the pre-operative CT scan.

**Figure 2:**
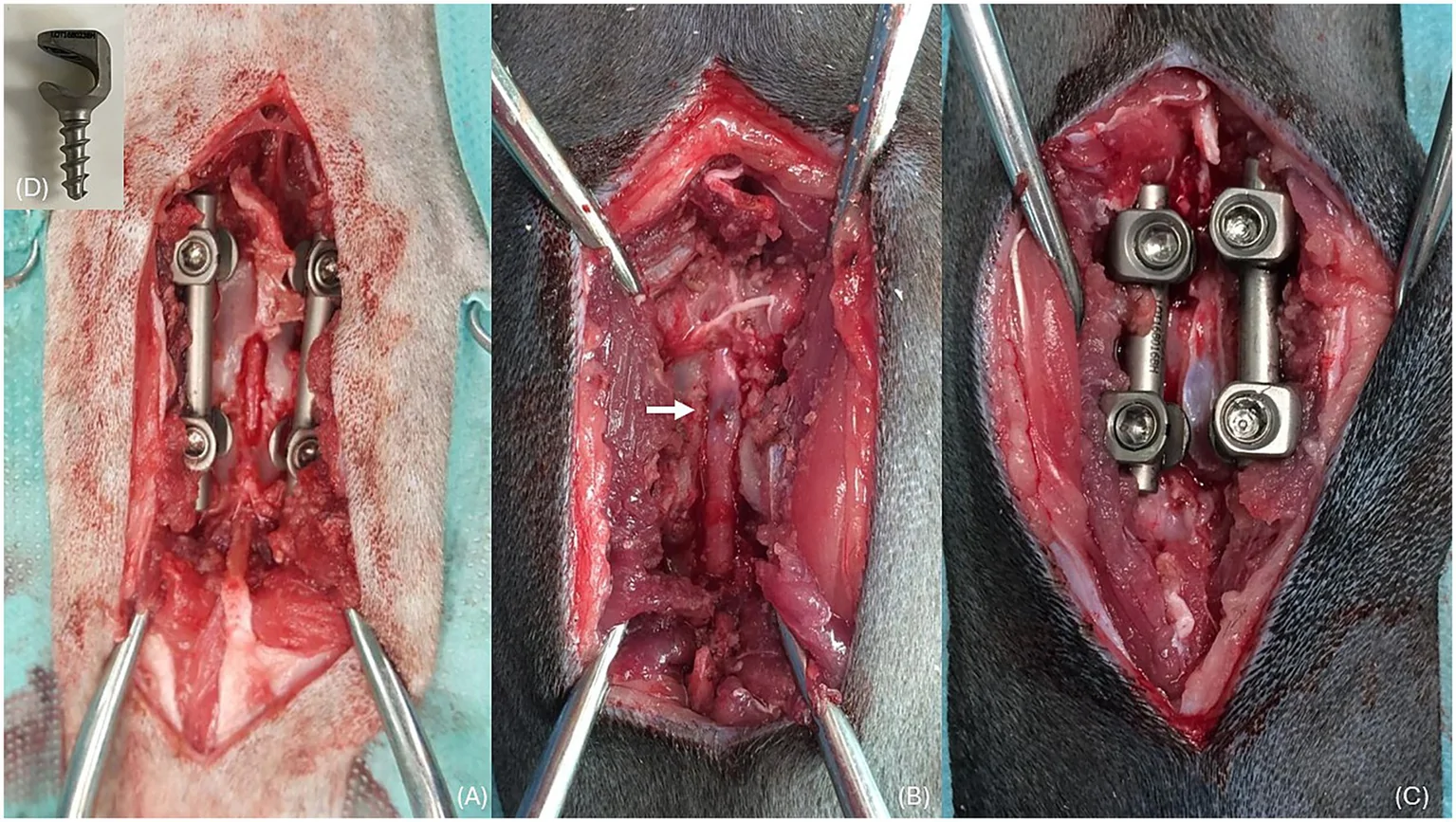
Intra-operative pictures of all cases. Orientation: cranial is at the top and caudal is at the bottom of the images. **(A)** Case 1 perioperative after PSRF placement. **(B)** Case 2 perioperative, arrow points to the darkened spinal cord. **(C)** Case 3 perioperative after PSRF placement. **(D)** Example of a pedicle screw. Core diameter: 2.0mm. Threat diameter: 2,5mm.

### 2.1 Case 1

A 1 year and 3 month old, 3.5 kg, spayed female European Shorthair cat, presented to the Utrecht University Small Animal Teaching Hospital (SA-TH) following trauma of unknown origin. On presentation, the cat was paraparetic with intact deep nociception (neurological grade III) according to the modified Griffith Sharp–Wheeler scale (24). After initial stabilization with intravenous fluid resuscitation and analgesia (boluses of 10 mL/kg isotonic saline IV in 15 min to effect on pulse rate and strength) and analgesia [meloxicam 0.2 mg/kg SC (Metacam, Boehringer Ingelheim) and methadon 0.2 mg/kg IV (Comfortan, Eurovet Animal Health BV)], lateral and dorsoventral radiographs of the lumbar spine and pelvis under minimal manual restraint were obtained. Radiographic examination (Figures 3A,B) revealed a comminuted fracture of the right caudal aspect of L6 with luxation of the articular facet joints and axial rotation. Given the often complex nature of spinal fractures and the need for accurate pre-operative measurements for implant sizing, a pre-operative computed tomography (CT by Philips Secura, Philips, NV, Eindhoven, the Netherlands) scan was discussed with the owners.

**Figure 3:**
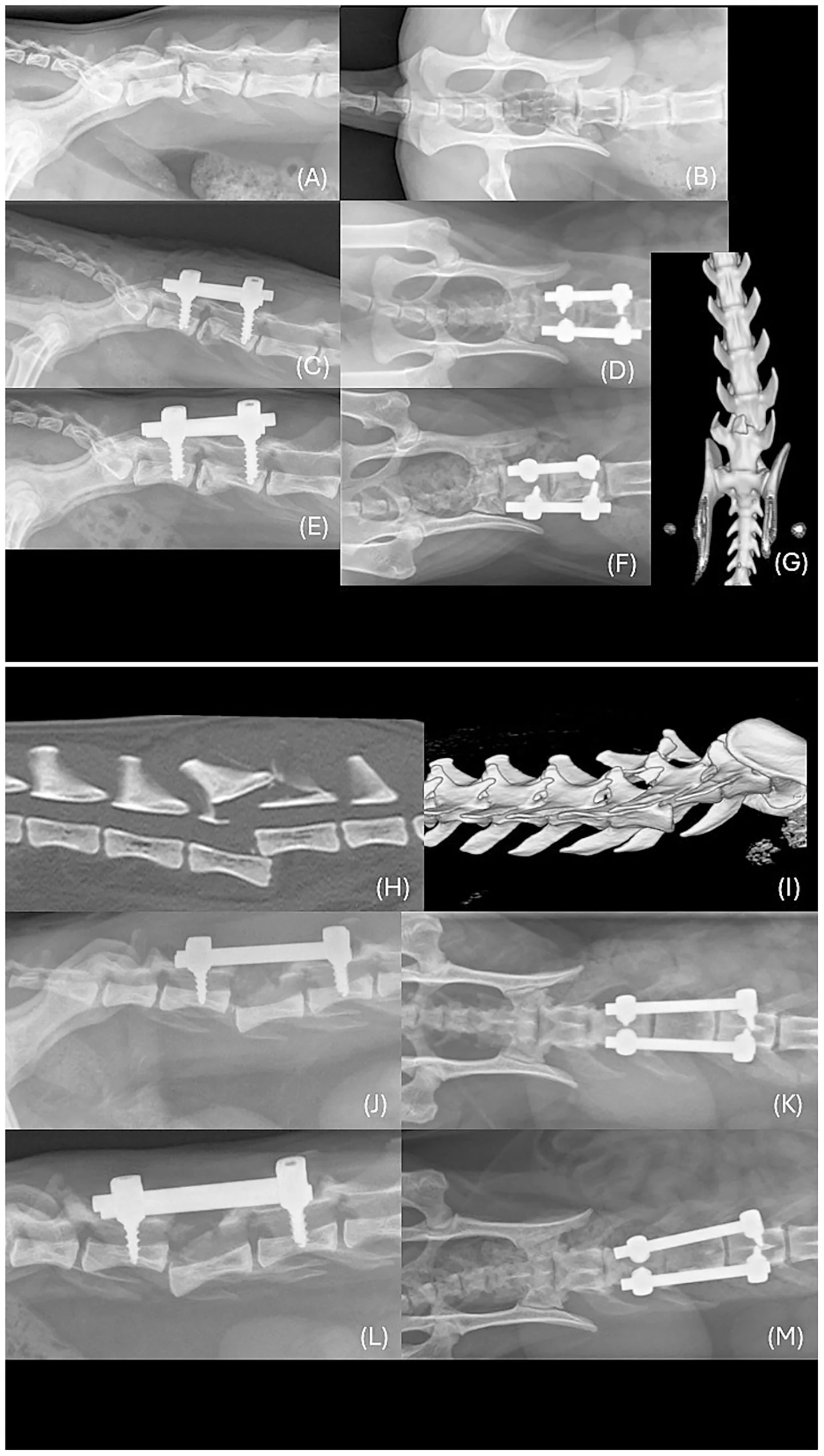
Case 1 and 3. Radiographs and CT. **(A)** Pre-operative right lateral radiograph case 1. **(B)** Pre-operative ventrodorsal radiograph case 1. **(C)** Post-operative right lateral radiograph case 1. **(D)** Post-operative ventrodorsal radiograph case 1. **(E)** 7 weeks post-operative right lateral radiograph case 1. **(F)** 7 weeks post-operative ventrodorsal radiograph case 1. **(G)** 3D reconstruction of the pre-operative CT scan case 1. **(H)** Pre-operative longitudinal CT scan case 3. **(I)** 3D reconstruction of the pre-operative CT scan case 3. **(J)** Post-operative right lateral radiograph case 3. **(K)** Post-operative ventrodorsal radiograph case 3. **(L)** 6 weeks post-operative right lateral radiograph case 3. **(M)** 6 weeks post-operative ventrodorsal radiograph case 3.

The day following initial presentation, the cat was anesthetized for advanced imaging and surgical intervention. Premedication consisted of methadone (0.2 mg/kg IV) and midazolam (0.2 mg/kg IV, Dormazolam, Dechra), followed by induction with propofol (1 mg/kg IV to effect, Proposure Multidose, Zoetis). Anesthesia was maintained using isoflurane inhalation. Perioperative analgesia included constant rate infusions (CRI) of ketamine (2–5 μg/kg/min, Anesketin, Dechra) and sufentanyl (0.2–0.5 μg/kg/h, Hameln). Perioperative antibiotic prophylaxis was provided with cefazoline (20 mg/kg IV, Cefazoline Mylan). Anticholinergics were administered if needed. Intraoperative monitoring included electrocardiography, capnography, pulse oximetry, and non-invasive arterial blood pressure measurement.

A CT scan (Philips Secura, Philips, NV, Eindhoven, the Netherlands) was performed with exposure settings of 120 kV and 260 mA, a pitch of 0.5, and a scan time of 1 s/rotation. Transverse images and reconstructed sagittal images were analyzed with the use of CT computer software (Philips CT Secura, Philips). The CT scan (Figure 3G) confirmed the radiographic findings, resulting in marked vertebral stenosis and compression of the spinal cord and cauda equina. The cat was positioned in sternal recumbency, and the surgical field was aseptically prepared using a chlorhexidine-alcohol protocol. A dorsal midline incision from the spinal process L5 to the sacrum was made using scalpel blade 10. Hemostasis was performed by bipolar electrocoagulation. The subcutis was dissected to expose the lumbosacral fascia. An incision was made paramedian of both spinal processes. The musculus longissimus dorsi and the musculus multifidi were dissected from the spinal process and from the articular facets by a periosteal elevator and Metzenbaum scissors. The dorsal lamina was cleared from any soft tissue attachments, enabling the placement of the pedicle screws into the vertebral pedicles after initial decompression. A limited dorsal laminectomy using a high-speed burr and Kerrison rongeurs and distraction were performed. The spinal cord showed a small indentation and mild blue discoloration at the level of the lumbar fracture and luxation.

Subsequently, four paired 3.5 mm monoaxial pedicle screws (Vertebral-Fix, Orthopedaesian) were placed into the vertebral pedicles. Screws measuring 16 mm in length were placed bilaterally in L6, and 14 mm screws were placed bilaterally in L7. Minimal breach of the medial cortices was detected by palpation in all four screws. The spinal cord was visible during screw placement through the dorsal laminectomy, and no damage of neural tissue was observed. Thereafter, 30 mm long, 4.0 mm rods were sideloaded bilaterally into the tulips of the pedicle screws and secured under distraction, with the objective of restoring spinal alignment (Figure 2A). An autologous fat graft harvested from the subcutis was placed over the surgical site, and the wound was closed in three layers. The fascia was closed by intermittent polydioxanone USP (PDS) 3–0. The subcutis was closed continuous with Poliglecaprone USP (Monocryl) 3–0. The cutis was subdermal closed continuously with Monocryl 3–0. Post-operative radiographs were made (Figures 3C,D).

Post-operatively, the patient was hospitalized for intravenous (IV) fluid therapy and continued analgesic management. Immediately post-operative, the cat was willing to stand and walk cautiously with paresis of the hindlimbs [neurological grade II, according to Wheeler and Sharp (24)]. The cat was discharged 2 days post-operatively once comfortable on oral pain medication. Oral medication consisted of a nonsteroidal anti-inflammatory drug (meloxicam 0.05 mg/kg orally SID, Metacam, Boehringer Ingelheim) for 2 weeks and buprenorphine [15 μg/kg transmucosal BID to TID, Buprenorphine (0.3 mg/mL buprenorphine buccal gel, magistral preparation by the Veterinary Pharmacy of the faculty of Veterinary Medicine Utrecht, The Netherlands)] for 2–3 days post operative. Strict activity restriction was advised, including no jumping, running, playing and stair climbing. At discharge, the cat was continent and ambulatory with mild paraparesis of the pelvic limbs and an impaired muscle tone of the tail, corresponding to neurological grade II (24).

The cat was evaluated during an in-hospital follow up 7 weeks post-operatively and showed no neurological deficits (grade 0) according to the Griffith Sharp–Wheeler scale (1994). Proprioception and peripheral reflexes were normal. The cat was not receiving any analgetic medication and was freely ambulatory within the house. Although slightly hesitant to jump, the cat was able to do so. Post-operative radiographs demonstrated stable implant presentation with new bone formation at the fracture site (Figures 3E,F). According to the owners, the cat remained neurologically normal for the subsequent 2 years following surgery, after which it ran away from home and was lost to follow-up.

### 2.2 Case 2

An 8-month-old, 3.4 kg, intact female Domestic Shorthair was admitted to the hospital following trauma of unknown origin after being missed overnight, 2 days prior to presentation. On neurological examination, voluntary movement and a withdrawal reflex were present in the right pelvic limb but absent in the left pelvic limb, corresponding to a neurological grade III for the right pelvic limb and grade IV paraplegia of the left pelvic limb (24). The anal reflex was absent, and tail tone was flaccid with absent deep nociception. Lateral and dorsoventral radiographs of the lumbar spine and pelvis were obtained under minimal manual restraint and confirmed a fracture of the vertebral body of L7. A pre-operative CT scan (Philips Secura, Philips, NV, Eindhoven, the Netherlands with identical exposure settings as used in case 1) (Figures 1C,D,I) demonstrated a compressive fracture of the L7 vertebral body, with multiple small fracture fragments present within the right intervertebral foramen and along the left ventrolateral aspect of the vertebra. Stenosis of the vertebral canal with moderate to severe cauda equina compression was observed. Surgical intervention was discussed with the owners, and the cat underwent surgery 5 days after the initial traumatic event. The delay in surgical intervention was due to the weekend and was deemed acceptable given the cat’s stable neurological status at the time of admission.

The patient was anesthetized using the protocol described in case 1, positioned in sternal recumbency, and the surgical field was prepared as previously described. A dorsal midline skin incision was made from the spinal process of L6 to the caudal medial sacral crest using a scalpel blade 10. Hemostasis was achieved by bipolar electrocoagulation. The subcutis was dissected to expose the lumbosacral fascia, followed by a bilateral paramedian incision adjacent to the spinal processes. The longissimus dorsi and the multifidi muscles were dissected from the spinal process at L6–L7 and L7–S1 facet levels using a periosteal elevator, bipolar device, and Stevens scissors. The spinous processes were removed by a standard rongeur, and a dorsal laminectomy was performed using a high-speed burr and Kerrison rongeurs; mild venous bleeding was noted. An intradural hematoma was visible at the cranial margin of the laminectomy, and the laminectomy was extended cranially where the spinal cord appeared darkened (Figure 2B). No marked bulging, bumps or loose fragments were observed ventrally in the spinal canal. The right L7 nerve root was notably swollen. Four paired 3.5 mm monoaxial pedicle screws (Vertebral-Fix, Orthopedaesian), each 16 mm in length, were placed in L7 and S1 vertebrae just caudal to the articular joints, as described by Lewchalermwong et al. (13). The spinal cord was visible during screw placement through the dorsal laminectomy, and no neural damage was observed during placement. Bilateral 4.0 rods, 40 mm in length, were contoured caudally to accommodate the vertebral anatomy, and secured under distraction resulting in a reduction of dorsal displacement of the caudal L7 and restoration of the vertebral canal space (Figure 1E). Perioperative fluoroscopy confirmed correct implant positioning and fracture realignment. An autologous fat graft harvested from the subcutis was placed, and the wound was closed in three layers similar to case 1. Post-operative radiographs were obtained (Figures 1E, F). At discharge 2 days post-operatively, the neurological examination findings were similar to those pre-operatively: neurological grade III for the right pelvic limb and grade IV paraplegia of the left pelvic limb (24). The cat was discharged with the same analgesic protocol as case 1.

The cat presented for a recheck 1 week post-operatively. Neurological function had improved, with voluntary movement and a positive withdrawal reflex in the previously non-functional left pelvic limb. Overall, a clear improvement in posture and limb movement was observed. However, the anal reflex, tail tone and nociperception in the tail remained absent.

At 6 weeks post-operative the cat was evaluated at an in-hospital follow up, case 2 remained incontinent and exhibited persistent tail paralysis. The pelvic limbs were used for ambulation, although paresis was still evident, corresponding to a neurological grade III (24). Lateral and dorsoventral radiographs obtained (Figures 1G,H) 6 weeks after surgery demonstrated stable implant positioning and evidence of fracture remodeling. Fracture fragments remained aligned, and no step deformity of the L7 vertebra was observed. The L7 vertebra displayed irregular radiopacity consistent with bone remodeling.

The second case was euthanized 6 months after surgery due to ethical concerns of the owner.

### 2.3 Case 3

A 6 year and 7 month old, 4.3 kg, neutered male European Shorthair cat was presented to the hospital following high-rise syndrome after which the owners reported that the cat was non-ambulatory. On presentation, the cat was paraplegic with present deep nociception of the pelvic limbs, consistent with a neurological grade IV (24). Voluntary movement was observed in the distal tail, and it was mildly painful to palpation. In this third case, lateral and dorsoventral radiographs of the lumbar spine and pelvis (obtained under minimal manual restraint), revealed a complete luxation of L5-L6, accompanied by multiple fractures and displaced fracture fragments. One day later, the neurologic grade deteriorated to grade IV (24). A pre-operative CT scan (Philips Secura, Philips, NV, Eindhoven, the Netherlands with identical exposure settings as used in case 1) (Figures 3H,I) confirmed the radiographic findings, resulting in displacement of the vertebral canal at the L5–L6 level without clear delineation of the spinal cord. Swelling and findings consistent with hemorrhage were present within and surrounding this level. Surgical intervention was discussed with the owners, and the cat was scheduled for surgery 1 day after initial presentation.

The patient was anesthetized using the protocol described in case 1, after which the cat was positioned in sternal recumbency and the surgical field was prepared as previously mentioned. A dorsal midline incision from the spinal process of L2 to L5 was made using scalpel blade 10. Hematoma formation was present in the subcutaneous tissue and beneath the fascia. Hemostasis was performed by bipolar electrocoagulation. The subcutis was dissected up to the lumbosacral fascia. A bilateral paramedian incision was made next to the spinal processes. The musculus longissimus dorsi and the musculus multifidi were dissected from the spinal process of L3 to L7 and from the articular facets by a periosteal elevator, bipolar device and Stevens scissors. A dorsal laminectomy was performed using a high-speed burr and Kerrison rongeurs, revealing a markedly swollen and hyperemic spinal cord. Spinal alignment was severely abnormal, the dura was ruptured, and the parenchyma was protruding. Reduction of the luxation was achieved using cranial and caudal distraction. Monoaxial pedicle screws (Vertebral-Fix, Orthopedaesian) were placed in L4 and L6 vertebral pedicles, just caudal to the articular facets. The spinal cord was visible when placing the screws through the dorsal laminectomy, and no damage of neural tissue was observed during placement (Figure 2C). Screws measuring 3.5 mm in diameter and 12 mm in length were used adapting to the size of the vertebra of the cat. An attempt was made to place pedicle screws in L5, however insufficient bone stock precluded successful placement. Bilateral 4.0 mm rods measuring 50 mm in length were placed and secured under distraction using Gelpi retractors, with the objective to restore spinal alignment. Peri-operative fluoroscopy showed acceptable implant placement but poor fracture realignment. An autologous fat graft harvested from the subcutis was placed in the osseous surgical defect within the dorsal lamina, a local splash block with morphine (0.2 mg/kg, Centrafarm) was placed, and the wound was closed in the same fashion as described for case 1. Post-operative lateral and dorsoventral radiographs were made (Figures 3J,K). The neurological grade immediately post-operatively was V (24). Post-operative care was similar to case 1. At discharge 8 days post-operatively, the cat was still incontinent and non-ambulatory, but showed subjective, subtle improvement in pelvic limb muscle tone, still corresponding to neurological grade V according to Wheeler and Sharp (24).

The cat was evaluated at three and 6 weeks post-operatively in-hospital. No neurological improvement was observed; the neurological grade remained V (24), with persistent paralysis posterior and urine incontinence. The tone and the voluntary movements of the tail and the tone of the anus were present. The spinal reflexes of the pelvic limbs remained intact. Radiographs obtained 6 weeks post-operatively demonstrated incomplete reduction of the luxation with worsened ventral luxation of L5 compared to L6 (Figures 3L,M). The implant presentation was stable. The cat did not regain pelvic limb voluntary movement or deep pain sensation and remained neurological grade V during a 6-month follow up period.

## 3 Discussion

This report describes the outcome of three cats with caudal lumbar fracture and luxation treated with pedicle screw rod fixation. All affected cats had a follow up period of at least 6 months. Two cats demonstrated neurological improvement, one of which improved from neurological grade III to grade 0 (24) and remained stable over a 2 year follow up period.

Vertebral fractures alone, or in combination with luxations, can cause spinal cord injuries in cats and dogs. Surgical treatment aims to decompress the spinal cord, and to stabilize and restore alignment of the vertebral column. To the authors’ knowledge, the use of bilateral pedicle screw rod fixation for caudal lumbar and lumbosacral fractured or luxated vertebrae had not yet been reported in cats.

The use of PSRF in cats has recently been reported for the thoracolumbar region (16, 17), but not for caudal lumbar and lumbosacral region. Only very recently a cadaveric study was reported describing a lumbosacral junction probing technique for implant corridors in cats (18). Çetin and Şirin (16) described the use of PRSF in cats with vertebral fractures and luxations that were treated with polyaxial screws unilateral in 13 cases and bilateral in three cases. 44 out of 50 polyaxial screws were placed optimally and 11 of the 13 cases showed neurological improvement. The feasibility and accuracy of vertebral pedicle screws in cats using a custom 3D printed drill guide and 3D-printed vertebral columns is described by Rigo et al. (17). Several alternative fixation techniques have been described in cats, including PMMA fixation (5). In dogs and humans, PSRF has been used successfully to treat degenerative lumbosacral stenosis (12–14).

To date, no consensus exists regarding treatment of caudal vertebral fractures and luxations in cats. The pedicle screw rod system fix was originally designed for human vertebrae. Meij et al. (21) reported that screws for pediatric patients are more suitable for canine vertebrae. A cadaver study using canine specific PSRF implants demonstrated improved implant placement in dogs, as these implants better accommodate the specific anatomy of the canine spine (13). It needs to be acknowledged that the use of relatively large implants in cats may cause catastrophic iatrogenic damage if planning is inadequate. Thorough pre-operative surgical planning is therefore essential. Although cats are not simply small dogs, the smallest dog-specific pedicle screws were successfully used in all three cats in this case series. The only available PSRF systems at the time of patient presentation (2016–2018) were a human monoaxial system with 4.2 pedicle screws (DePuy Synthes, Universal Small Stature) and a veterinary system from Thailand (Vertebral-Fix, Orthopedaesia), with 3.5 monoaxial pedicle screws. The 4.2 screws were considered not appropriate for cats. Precise measurement using a digital caliper of the V-Fix 3.5 screws showed a screw shaft core of 2.5 mm and diameter of the screw thread of 3.5 mm (Figure 2D). The pedicle screw core tapered to a diameter of 2.0 mm at the end (Figure 2D). The rods had a diameter of 4.0 mm. Based on the pre-operative measurements it was concluded that the screws were considered safe to use in the cats, although we expected there would be encroachment to some frank penetration of the pedicle walls at its most narrow point (14). As all cases in this report had a dorsal laminectomy performed prior to PSRF placement, the breach of the medial wall and its relation to the neural structures within the spinal canal could be monitored readily. As species-specific implants become increasingly available, it is anticipated that PSRF systems will become more widely integrated into spinal surgery for cats with surgical spinal diseases.

According to Vallefuoco et al. (25), screw diameter for cats should not exceed 2 mm to allow safe placement. Given that thoracic and lumbar vertebrae in cats are relatively small, adherence to defined safe corridors for implant placing—such as screws and pins—is essential to avoid iatrogenic injury to neural and vascular structures, especially when placed without direct vision into the spinal canal. Recently described optimal safe corridors in cats allow maximal bone purchase for bicortical implants while improving surgical safety. At the time these cases were treated (2016–2018), the smallest available implants measured 3 mm in diameter, exceeding the 2 mm recommended for cats (5). Currently, smaller screws of 2.0 and 2.7 mm, designed for small dogs and cats, are entering the veterinarian market. However, breaching of the medial cortex of the pedicle does not imply inadvertent neurogenic trauma. Intraoperative palpation of the glide hole, or even direct visual inspection may indicate cortical breach. However, screw placement can proceed if the breach is minimal and causes no or minimal spinal canal narrowing.

A lack of standardization regarding vertebral safe corridors for the use of these 3.5 mm pedicle screw rod fixation system in cats should be noted. Pre-operative CT images were used for measuring the safety corridors for implant sizing. In all cases, though the implants are considered oversized in the current market, placement was deemed safe by the surgeons involved in these cases after measuring the screw corridors. The alternatives to spinal stabilization at the time (2016–2018) were also limited in their success (6–10, 34). With the experience with PSRF in spinal stabilization in dogs at our hospital, offering this to feline patients was considered reasonable and justified. In the veterinary market, more appropriated sized implants are becoming more available. Measuring the size of the screw corridor in the pedicles in the individual patient (Table 1, Figures 1A,B) should be performed for optimal implant sizing. Nowadays the recommendations given by Rigo et al. (17) could be used while considering placement of pedicle screws in the thoracolumbar spine of cats. The recommendations described by Rigo et al. (17) for the caudal lumbar spine (up to L7) and the probing technique for the lumbosacral corridors by Mullins et al. (18) should be considered for placement of pedicle screws in the caudal lumbar and lumbosacral spine of cats.

According to the literature, indications for surgical intervention in vertebral fractures and luxations in cats remain a topic of debate, but surgery could be considered in cats presenting with paraparesis and intact nociception. There are very few studies on the outcome of spinal surgery in cats in general and most surgeons are forced to extrapolate the outcomes from pet dogs or even human surgery to their feline counterparts (26–31). The prognosis seems to be related to deep pain perception pre-operatively. Limitations of this study include the extremely small sample size. As the use of PSRF for the treatment of vertebral fractures and luxations becomes more widespread, it is anticipated that a larger caseload will be available for analysis. Increased case numbers may allow for the identification of prognostic factors associated with PSRF outcomes in cats. Further investigation of treatment outcomes, particularly comparisons between neurological grade at presentation and post-operative neurological grade, may aid in the development of more standardized treatment recommendations for this condition as multiple factors contributes to neurological recovery following vertebral spinal trauma. Another limitation of this case series is that procedures were not performed by a single surgeon with potential variability in surgical technique. However, all surgeries were conducted at the same university teaching hospital by board-certified surgical specialists, all of whom were trained by the same surgeon (BM).

Time can be a critical prognostic factor in cases with severe fracture-luxation and absent deep pain perception. The delayed surgical intervention in this case series was caused by patient dependent issues, including a delay in presentation after trauma (case 2), owner decision making, advanced diagnostic imaging and availability of the surgical team. Constructing a well-equipped surgical team and surgical strategy consumes time as these cases do require a certain amount of experience and surgical dexterity, which is not always present after hours. Delaying surgical intervention may therefore be necessary to improve patient outcome, however time is of essence, especially in unstable fractures (32, 33). The availability of advanced diagnostic imaging and knowledgeable surgeons out of hours in veterinary medicine however, may be a limiting factor for our veterinary patient. Other limitations of this case series include the lack of a standardized outcome assessment, as well as the absence of a standardized complication classification, both are due to theretrospective nature with variable follow-up durations. Overall, there is a lack of standardization regarding safe vertebral corridors in the cat and further long-term comparative studies are required to adequately assess technique-related complications.

Two of the three cases described in this report demonstrated neurological improvement following PSRF surgery, without implant associated complications. In the third case, which did not show neurological improvement, incomplete reduction of the L5 luxation and severe spinal cord damage observed intra-operatively could likely explain the absence of clinical improvement. The other two cases outline the potential of PSRF as a treatment option for cats with caudal vertebral fractures and luxation. However, case selection and outcome need to be interpreted cautiously, and further investigated to draw consistent prognostic values and conclusions regarding this intervention. Interpretation of neurological improvement in our cases should be cautious, as outcomes may primarily reflect initial injury severity rather than surgical technique.

This case series demonstrates the feasibility of treating cats with fractures and luxations using a monoaxial PSRF system. PSRF is a promising surgical option for the treatment of vertebral luxations and fractures in cats, however, its application warrants further investigation.

## Data Availability

The original contributions presented in the study are included in the article/supplementary material, further inquiries can be directed to the corresponding author.
